# Green Synthesis of Zinc Oxide Nanoparticles for Tetracycline Adsorption: Experimental Insights and DFT Study

**DOI:** 10.3390/plants13233386

**Published:** 2024-12-02

**Authors:** Solhe F. Alshahateet, Salah A. Al-Trawneh, Mohammed Er-rajy, Mohammed Zerrouk, Khalil Azzaoui, Waad M. Al-Tawarh, Belkheir Hammouti, Rachid Salghi, Rachid Sabbahi, Mohammed M. Alanazi, Larbi Rhazi

**Affiliations:** 1 Department of Chemistry, Faculty of Science, Mutah University, Mutah, Karak 61710, Jordan; laratr@mutah.edu.jo (S.A.A.-T.); waaltawarh@gmail.com (W.M.A.-T.); 2 LIMAS Laboratory, Faculty of Sciences Dhar El Mahraz, Sidi Mohamed Ben Abdellah University, Fez 30000, Morocco; mohammed.errajy@usmba.ac.ma; 3 Engineering Laboratory of Organometallic, Molecular Materials and Environment, Faculty of Sciences, Sidi Mohamed Ben Abdellah University, Fez 30000, Morocco; zrkmed0650@gmail.com (M.Z.); k.azzaoui@yahoo.com (K.A.); 4 Euromed Research Center, Euromed Polytechnic School, Euromed University of Fes, UEMF, Fes 30030, Morocco; hammoutib@gmail.com; 5 Laboratory of Industrial Engineering, Energy and the Environment (LI3E) SUPMTI, Rabat 10000, Morocco; 6 Laboratory of Applied Chemistry and Environment, ENSA, University Ibn Zohr, P.O. Box 1136, Agadir 80000, Morocco; r.salghi@uiz.ac.ma; 7 Research Team in Science and Technology, Higher School of Technology of Laayoune, Ibn Zohr University, Laayoune P.O. Box 3007, Morocco; 8 Department of Pharmaceutical Chemistry, College of Pharmacy, King Saud University, Riyadh 11451, Saudi Arabia; mmalanazi@ksu.edu.sa; 9 Institut Polytechnique UniLaSalle, Université d’Artois, ULR 7519, 19 rue Pierre Waguet, BP 30313, 60026 Beauvais, France; larbi.rhazi@unilasalle.fr

**Keywords:** biosynthesis, nanoparticles, ZnO NPs, *Thymus vulgaris*, adsorption, tetracycline, kinetic, isotherm, thermodynamic

## Abstract

An eco-friendly approach was used to fabricate zinc oxide nanoparticles (ZnO NPs) using thyme, *Thymus vulgaris* L., leaf extract. The produced ZnO nanoparticles were characterized by XRD and SEM analysis. The ZnO NPs showed remarkable adsorption efficiency for tetracycline (TC) from water systems, with a maximum removal rate of 95% under optimal conditions (10 ppm, 0.10 g of ZnO NPs, pH 8.5, and 30 min at 25 °C). The adsorption kinetics followed the pseudo-2nd-order model, and the adsorption process fitted the Temkin isotherm model. The process was spontaneous, endothermic, and primarily chemisorptive. Quantum chemistry calculations, utilizing electrostatic potential maps and HOMO-LUMO gap analysis, have confirmed the stability of the TC clusters. This study suggests that green synthesis using plant extracts presents an opportunity to generate nanoparticles with properties suitable for real-world applications.

## 1. Introduction

Conventional chemical methods for synthesizing beneficial compounds often need to be improved as they possess limitations such as poor control over particle size, high cost, hazardous by-products, and low yields. This highlights the need for innovative, eco-friendly approaches to develop novel materials with desirable properties for diverse real-world applications. Recently, biosynthesis has emerged as a green strategy for producing metal oxide nanoparticles (MO NPs) using naturally occurring biological entities, such as plant extracts, bacteria, and algae [1,2]. For instance, previous studies have shown that silver nanoparticles (Ag NPs) synthesized from plant extracts exhibit significantly lower cytotoxicity and phytotoxicity, indicating that green Ag NPs are safer and suitable for broader applications [3,4]. In another study, zinc oxide nanoparticles (ZnO NPs) were synthesized using mint leaf extract, which endowed the nanoparticles with favorable physical and chemical properties for medical applications [5]. Additionally, Fe_2_O_3_ NPs produced using cloves extract have shown potential for cancer treatment and water purification [6,7].

MO NPs are highly valued due to their nanoscale size and large surface area, which grant them unique properties distinct from their bulk forms. These nanoparticles exhibit diverse physical, chemical, biological, optical, magnetic, and catalytic characteristics, fueling significant research interest. They offer promising applications in biomedical, biosensors, catalysis, drug delivery, and cosmetics. The morphology of these particles dramatically influences their characteristics [8,9], and as a result, various synthesis methods have been explored extensively.

ZnO NPs are shared and widely utilized across many applications. Their unique physical characteristics, such as a large surface area, transparency, and conductivity, make them appropriate for biosensors, drug delivery, and electrical and optical devices [10]. ZnO NPs also exhibit high chemical and biological activity, enabling their use in medical applications like catalysts and cancer therapy, and environmental applications like water purification and air quality improvement. Further, they play a role in the technology sectors, particularly in semiconductors and solar power. Given their abundance and cost-effectiveness, ZnO NPs hold great promise for multiple applications, although their potential environmental and health impacts require careful monitoring.

The synthesis of metal oxide nanoparticles (MNPs) using thyme, *Thymus vulgaris* L., is of interest due to its rich phytochemical composition, which includes a wide range of bioactive compounds. These compounds play a crucial role in mediating the synthesis, stabilization, and reduction of metal ions to form metal oxide nanoparticles. A more specific breakdown of how these bioactive compounds can influence the synthesis of MNPs follows.

In the case of zinc oxide (ZnO) nanoparticles, thyme extract has been shown to reduce Zn^2+^ ions to ZnO nanoparticles in a one-step biosynthesis process. The phenolic and flavonoid compounds in thyme extract are believed to play the role of reducing agents, while terpenoids and other plant metabolites act as stabilizing and capping agents. This process results in ZnO nanoparticles with good dispersion, enhanced antibacterial properties, and potential applications in sensors, medicine, and catalysis.

The phytochemical composition of *T. vulgaris* directly influences the synthesis and properties of metal oxide nanoparticles. The polyphenolic compounds, terpenoids, and essential oils in thyme contribute to the reduction of metal ions, stabilization of the nanoparticles, and control over the size and shape of the particles. This makes *T. vulgaris* a promising candidate for the green synthesis of metal oxide nanoparticles with various potential applications in medicine, agriculture, and environmental remediation.

Adsorption is one of the leading water treatment techniques to remove organic and inorganic pollutants from drinking and industrial water. This process depends on materials, such as activated carbon, organic compounds, biochar, and silica gel [11,12,13,14,15], which attract and capture impurities on their surfaces. Adsorption is cost-effective, environmentally friendly, and capable of improving water quality without the use of harmful chemicals. As a critical process in water purification, adsorption contributes significantly to the availability of clean, drinkable water, enhancing public health and preserving ecosystems. The growing adoption of adsorption techniques underscores the ongoing global commitment to improving water quality in response to the needs of an expanding population.

## 2. Results and Discussion

### 2.1. Characterization

#### 2.1.1. X-Ray Diffraction

**Figure 1:**
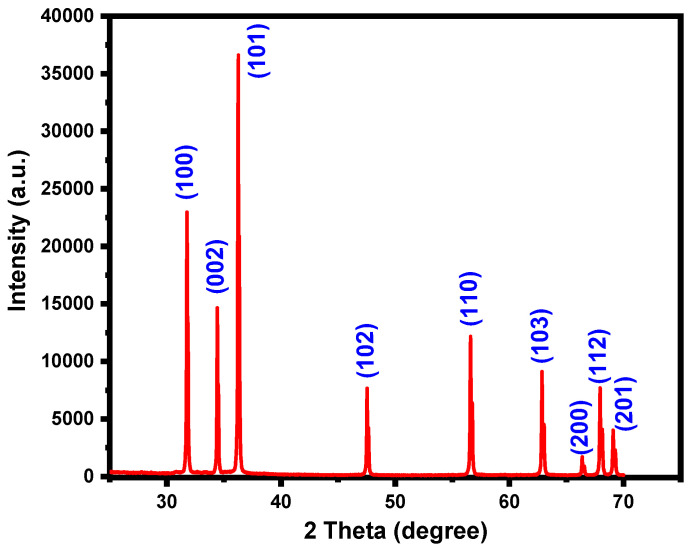
XRD pattern of ZnO NPs.

The structural and phase purity of ZnO nanoparticles is illustrated in Figure 1. The XRD diffractogram aligns well with the hexagonal wurtzite structure, as confirmed by comparison with JCPDS card No. 89-1397, showing no evidence of secondary phases or impurity peaks. The strong, narrow diffraction peaks indicate that the product has a high-quality crystalline structure. The sharp, intense diffraction peaks observed at approximately 2θ values of 31.29°, 33.95°, 36.04°, 47.05°, 56.09°, 62.38°, 65.90°, 67.45°, and 68.60° correspond to the crystal planes (1 0 0), (0 0 2), (1 0 1), (1 0 2), (1 1 0), (1 0 3), (2 0 0), (1 1 2), and (2 0 1), respectively.

#### 2.1.2. SEM Analysis

**Figure 2:**
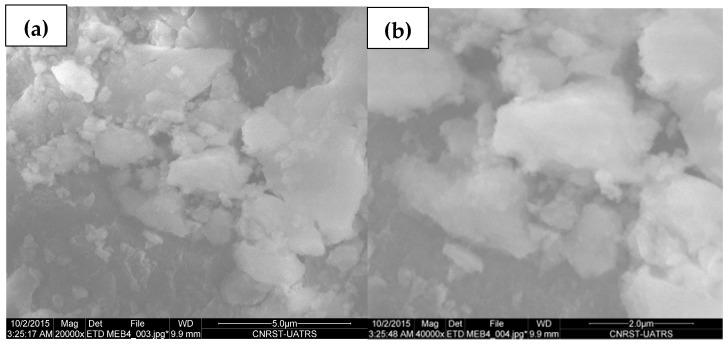
SEM image of synthesized ZnO NPs using thyme, *Thymus vulgaris* L., leaf extract, (**a**) ×20,000, (**b**) ×40,000.

The scanning electron microscopic (SEM) analysis in Figure 2 shows that ZnO nanoparticles have an agglomerated structural morphology. The compact morphology of agglomerated nanoparticles may be attributed to the use of heat in the electric furnace that imparts the crystalline nature and is responsible for the reorganization of the biomolecules responsible for the capping as well as the stabilization of the ZnO NPs synthesized using thyme, *Thymus vulgaris* L., leaf extract.

### 2.2. Quality Assurance

**Figure 3:**
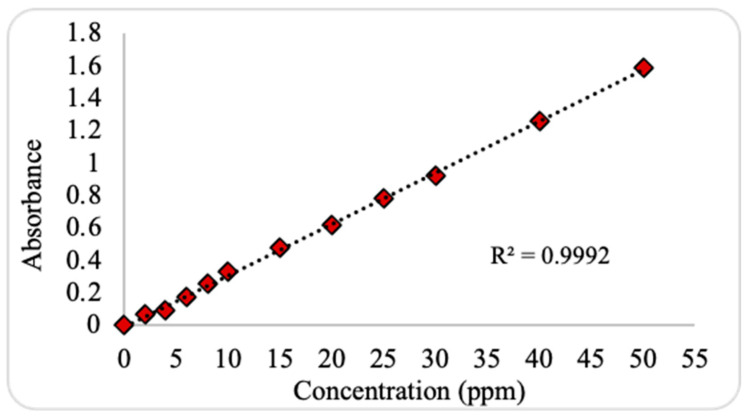
External calibration curve for the determination of tetracycline concentrations.

The calibration curve linearity was verified according to the correlation coefficient (R^2^), which exceeded 0.9992, as shown in Figure 3. The LOD was identified based on a signal-to-noise ratio of (3:1) and was 0.1309 ppm, with the SD value expressed as <2%. Similarly, the LOQ was identified by a signal-to-noise ratio of (10:1), and it was 0.3963 ppm. It can be noted that the LOQ was less than the lowest TC concentration obtained during the analysis (around 0.5321 ppm). Furthermore, an acceptable recovery rate of 97% ± 2 was achieved, which confirms the appropriateness of our laboratory analysis methodology.

### 2.3. Optimization of Specific Parameters on TC Adsorption

#### 2.3.1. Nanosorbent Dose Influence

**Figure 4:**
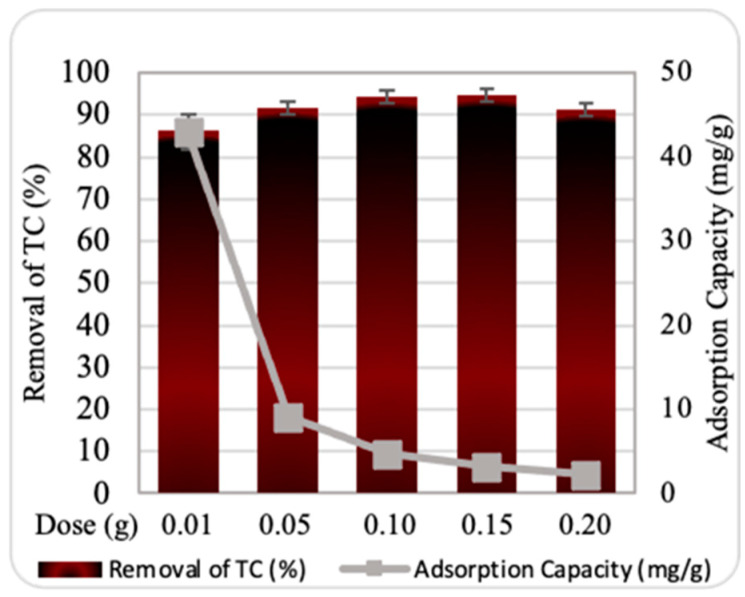
Impact of ZnO NPs dosage on removal efficiency and adsorption capacity. Experimental conditions: contact time, 30 min; starting tetracycline (TC) concentration, 10 ppm; temperature, 25 °C.

The effect of ZnO NPs dosage on TC adsorption efficiency and capacity was evaluated using a range of 0.10 to 0.20 g, with a starting TC concentration of 10 ppm, a contact time of 30 min, and a temperature of 25 °C. As shown in Figure 4, the maximum TC removal efficiency reached 94% at a ZnO NP dosage of 0.10 g. However, at higher ZnO NP dosages, the removal efficiency remained nearly constant or slightly decreased. This may be attributed to the nanosorbent’s large surface area, which provides ample adsorption sites even at lower dosages.

Moreover, the highest adsorption capacity of ZnO NPs was 42.9 mg/g at a dosage of 0.10 g. Notably, the adsorption capacity decreased significantly as the dosage of the nanosorbent increased. This trend could be explained by the presence of unsaturated active sites on the nano-surface at lower dosages. Once maximum adsorption is achieved, further increases in dosage may result in sorbent accumulation and competition between substances, leading to a decline in adsorption capacity.

#### 2.3.2. Starting TC Concentration Influence

**Figure 5:**
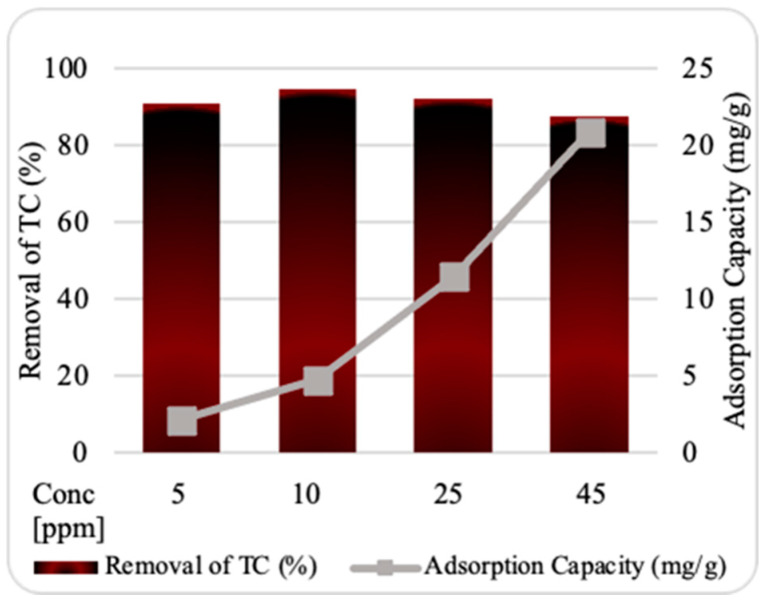
Impact of starting tetracycline (TC) concentration on adsorption by ZnO NPs. Experimental conditions: nanosorbent dosage, 0.10 g; contact time, 30 min; pH, 7.6; temperature, 25 °C.

The effects of the initial amount of TC on the removal percentage by ZnO NPs were investigated at 5, 10, 25, and 45 ppm of TC, as shown in Figure 5. The maximum adsorption efficiency during the batch experiment duration was obtained with a starting TC concentration of 10 ppm. The results indicate that adsorption at low concentrations may be highly driven by the combination of the high concentration of the adsorbate at the initial stage and the large number of readily available adsorption sites. As the TC molecules start competing for the remaining sites and the adsorbed ZnO NPs begin to generate repulsive interactions during further adsorption, the number of available sites may gradually decrease [16], causing low adsorption efficiency. In contrast, the adsorptive capacity of the prepared nanosorbent was found to increase dramatically as the TC concentration increased, suggesting the good adsorptive ability of ZnO NPs towards TC, as described in previous literature [14].

#### 2.3.3. Contact Durations Influence

**Figure 6:**
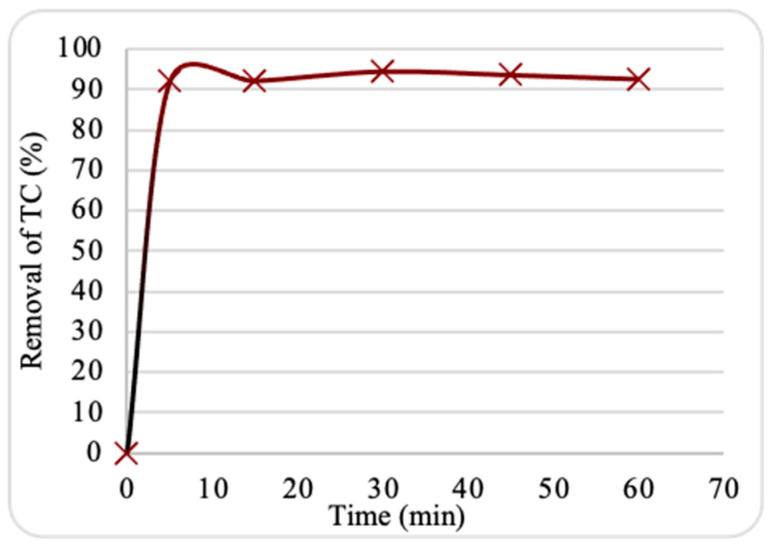
Impact of contact duration on tetracycline (TC) adsorption by ZnO NPs. Experimental conditions: nanosorbent dosage, 0.10 g; pH, 7.6; starting TC concentration, 10 ppm; temperature, 25 °C.

To discover the impact of contact duration on the adsorption removal of TC, batch experiments were performed with contact times ranging from 0 to 60 min. The removal efficiency of TC onto the prepared nanosorbent as a function of contact time is illustrated in Figure 6. The adsorption is a multistep process, with quick removal in the first 5 min and relatively gradual adsorption from 10 to 60 min. At 30 min, the TC removal rate reached equilibrium with a maximum removal of 95%. The results may be explained by the large quantity of vacant active sites and the appropriate pore size of the nanosorbent surface, which facilitate internal mass transfer and the progress of the adsorption process [17]. Nevertheless, due to the accumulation of TC molecules on the nanosorbent surface and an increase in internal diffusion resistance, the adsorption rate gradually decreased after 30 min. Overall, the findings suggest that the produced nanosorbent has a preference for adsorbing TC molecules and are in good agreement with previous results [15].

#### 2.3.4. pH Influence

Numerous studies have emphasized the substantial impact of solution pH on TC adsorption in aqueous media [14,16,18]. Determining the optimal pH is crucial for evaluating the interactions between a nanosorbent and TC ions about adsorption efficiency. Different pH levels may influence the surface charge of the nanosorbent, the distribution of TC species, and the degree of dissociation of functional groups on the active sites of TC ions.

Al-Tawarh et al., 2023 [14] suggested that at the point of zero charge pH (pH_PZC_), the adsorbent surface has no net charge at a specific pH value. In other words, the ZnO NP surfaces have an equal balance of negatively and positively charged groups. It is established that when the solution’s pH is below pH_PZC_, the nanosorbent’s surface is positively charged. Conversely, when the pH exceeds pH_PZC_, the surface of the nanosorbent becomes negatively charged [19]. Thus, understanding pH_PZC_ provides insight into how pH affects adsorption.

**Figure 7:**
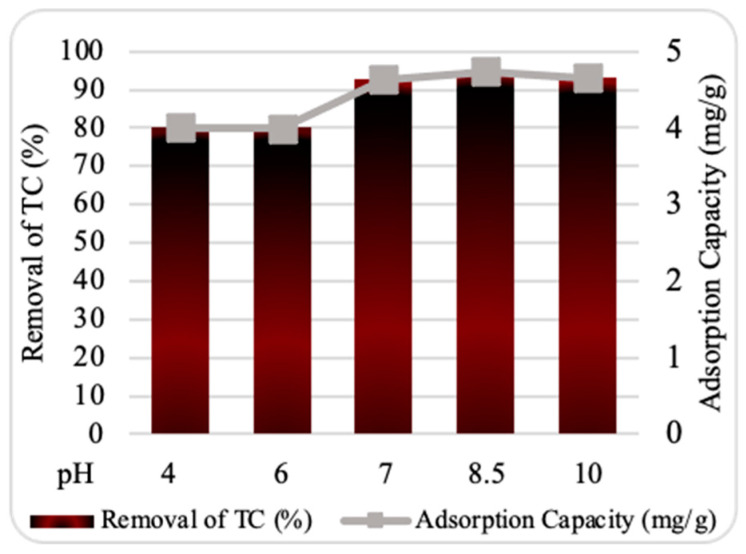
Effect of pH on tetracycline (TC) adsorption by ZnO NPs. Experimental conditions: nanosorbent dosage, 0.10 g; contact time, 30 min; starting TC concentration, 10 ppm; temperature, 25 °C.

The relation between the percentage of TC adsorbed at various pH levels is represented in Figure 7. The results show that the optimum adsorption of TC onto ZnO NPs occurred at pH 8.5, achieving a removal efficiency of 95%. This suggests that neutral pH values (~pH_pzc_) favor TC adsorption onto ZnO NPs. At pH < pH_pzc_, the abundance of protons (H^+^) competes with TC ions for active adsorption sites, decreasing TC adsorption. Conversely, at pH > pH_pzc_, the deprotonated forms of TC predominate, resulting in negatively charged surfaces that create electrostatic repulsion with the similarly charged TC ions, reducing adsorption [20]. Similar findings have been reported in studies on TC removal from aqueous solutions [21].

### 2.4. Effect of Temperature

**Figure 8:**
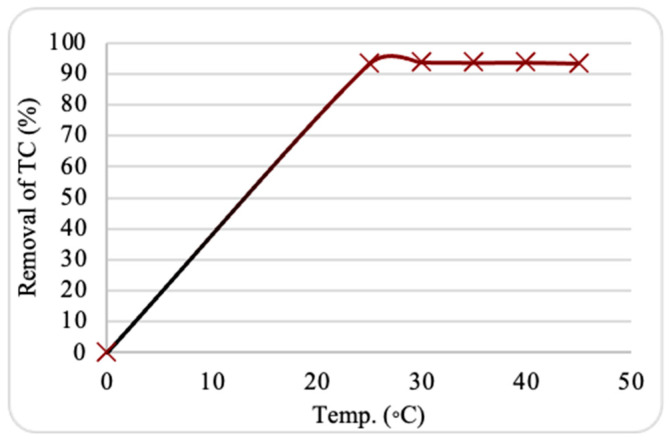
Impact of temperature on tetracycline (TC) adsorption by ZnO NPs. Experimental conditions: nanosorbent dosage, 0.10 g; pH, 8.5; contact time, 30 min; starting TC concentration, 10 ppm.

The effects of temperature on adsorption were examined over a range of 25–55 °C to assess the effectiveness of ZnO NPs further. Figure 8 shows that the maximum TC adsorption onto ZnO NPs occurred at 25 °C, with a removal of 95%. Adsorption efficiency reduced significantly as the temperature increased. This trend may be attributed to damage to some active sites due to the temperature rise, reducing the nanosorbent’s ability to bind with TC molecules. The solubility and mobility of TC may also reduce at high temperatures, causing reduced adsorption efficiency.

Thermodynamic experiments were performed to examine the relationship between adsorption capacity and temperature. The thermodynamic parameters, including thermodynamic equilibrium constant (K_d_, Equation (1)), enthalpy changes (ΔH^0^, Equation (2)), entropy (ΔS^0^, Equation (2)), and Gibbs free energy (ΔG^0^, Equation (3)), were calculated and are presented in Table 1. R and T represent the universal gas constant (8.314 J/mol·K) and temperature (K), respectively.

(1) $$K_{d}=\frac{q_{e}}{C_{e}}$$

(2) $$\ln K_{d}=\frac{∆S^{0}}{R}-\frac{∆H^{0}}{RT}$$

(3) $$∆G^{0}=∆H^{0}-∆S^{0}T$$

The values of the ΔG^0^, ΔS^0^, and ΔH^0^ presume that TC adsorption onto ZnO NPs is spontaneous, endothermic, and more disordered in nature, with the system tending to reach an equilibrium position.

### 2.5. Adsorption Kinetic onto ZnO NPs 

**Figure 9:**
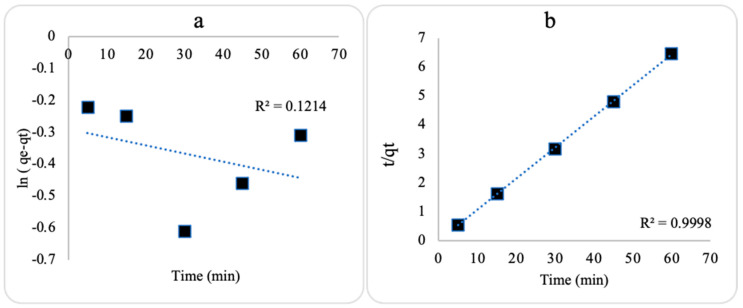
Kinetic plots of (**a**) pseudo-1st order and (**b**) pseudo-2nd order for tetracycline adsorption onto ZnO NPs.

The kinetics of TC adsorption were evaluated using the pseudo-1st-order (Equation (4)) and pseudo-2nd-order (Equation (5)) kinetic models. The adsorption amount in mg of TC per g of ZnO NPs at equilibrium (q_e_) and various contact durations (q_t_) was calculated (Table 2). The linearized plots of the kinetic models are represented in Figure 9. The level of linearity (R^2^) values indicated that TC adsorption onto each prepared ZnO NP followed a pseudo-2nd-order model. This model suggests the dominance of chemisorption behavior between TC molecules and ZnO NP surfaces. The kinetics results corresponded with previous literature data [16].

(4) $${ln(q}_{e}-q_{t})={lnq}_{e}-K_{1}t$$

(5) $$\frac{t}{q_{t}}=\frac{1}{K_{2}q_{e}^{2}}+\frac{t}{q_{e}}$$

### 2.6. Isotherm Parameters for the Adsorption onto ZnO NPs 

Adsorption isotherms were studied to assess TC distribution between the solid and aqueous phases as a function of TC concentration, the interaction between nanosorbents and TC, and the adsorption behavior (whether it involves monolayer or multilayer adsorption). The Langmuir, Freundlich, and Temkin isotherm models were applied using linear regression analysis, as represented by (Equation (6)), (Equation (7)), and (Equation (8)), respectively. All calculated isotherm parameters for TC adsorption are summarized in Table 3.

(6) $$\frac{C_{e}}{q_{e}}=\frac{1}{q_{max}K_{L}}+\frac{C_{e}}{q_{max}}$$

(7) $$\ln q_{e}=\ln K_{F}+\left(\frac{1}{n}\right)\ln C_{e}\;$$

(8) $$q_{e}=B_{T}\ln K_{T}+B_{T}\ln C_{e}$$

**Figure 10:**
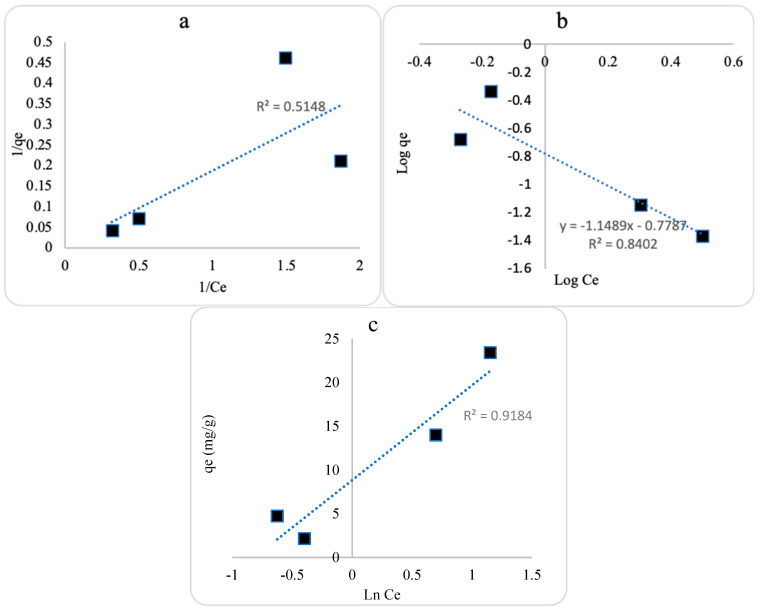
Isotherm plots of (**a**) Langmuir isotherm, (**b**) Freundlich isotherm, and (**c**) Temkin isotherm of tetracycline adsorption onto ZnO NPs.

As shown in Figure 10, the experimental data of TC adsorption onto ZnO NPs best fit the Temkin isotherm model based on R^2^ values. The higher B_T_ and K_T_ values indicate stronger adsorption interactions between ZnO NPs and TC, demonstrating that TC is more strongly attracted to the surface of ZnO NPs. Additionally, a higher concentration of TC on the surface of ZnO NPs reflects the level of favorability of TC adsorption [10,22].

### 2.7. Atomistic Models of the Adsorbent and Adsorbate

#### 2.7.1. Geometry Optimization

**Figure 11:**
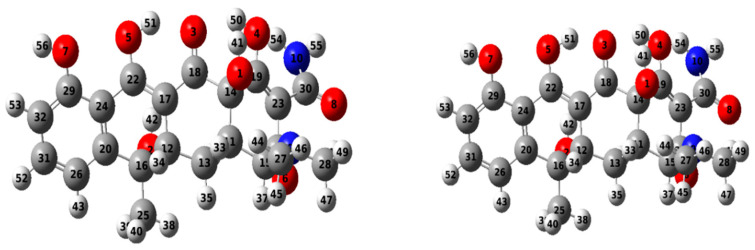
Optimized geometry of tetracycline, showing atom positions (red = oxygen, blue = nitrogen, grey = carbon).

The molecular geometry was optimized to assess its influence on the optoelectronic properties of TC. Calculations were performed at the DFT/B3LYB and 6–31G (d, p) basis set levels to optimize the ground state configuration of TC. Figure 11 presents the optimized geometry of TC with atom numbers.

#### 2.7.2. Frontier Molecular Orbital Analysis

**Figure 12:**
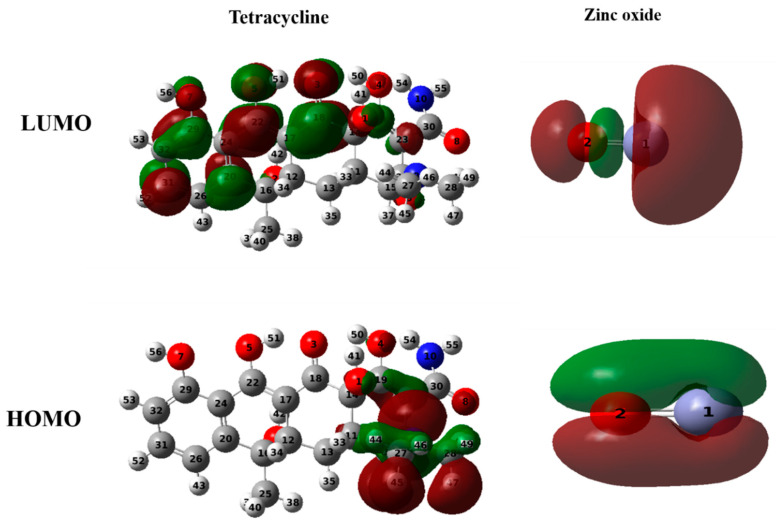
Frontier molecular orbitals of tetracycline and zinc oxide.

The Frontier molecular orbitals (FMOs), namely the Highest Occupied Molecular Orbital (HOMO) and the Lowest Unoccupied Molecular Orbital (LUMO), are crucial in determining molecular interactions, chemical reactivity, kinetic stability, and electrical and optical characteristics. So, FMO analysis was conducted to investigate the reactivity and electron distribution in the designed systems [23]. The calculated HOMO and LUMO energies of TC and zinc oxide are presented in Figure 12.

HOMO energy represents a molecule’s capacity to donate electrons, while LUMO energy corresponds to its ability to accept electrons. The energy gap between HOMO and LUMO plays a pivotal role in chemical reactivity, with a smaller gap indicating lower kinetic stability.

Figure 12 illustrates that HOMOs are located on the ring substituted by dimethylamine, while LUMOs are located on the oxygen side of the other ring atoms present in TC. We can, therefore, deduce that the dimethylamine molecule acts as an electron donor, while the ring oxygens present in TC act as electron acceptors. In the case of zinc oxide, both HOMO and LUMO are distributed across the entire oxide. This indicates that zinc oxide can act as an electron donor and acceptor. The electronic properties of TC and zinc oxide are summarized in Table 4.

The calculated LUMO and HOMO energies for TC were −2.73 and −5.50 eV, respectively (Table 4), resulting in a gap of 2.77 eV. This narrow gap suggests the presence of charge transfer interactions and indicates that TC is highly polarizable and reactive.

Similarly, the HOMO and LUMO energies for zinc oxide were calculated as −6.63 eV and −4.09 eV, respectively, with an energy gap of 2.53 eV. These results indicate that zinc oxide, like TC, is highly reactive due to its small energy gap.

#### 2.7.3. Molecular Electrostatic Potential Analysis

**Figure 13:**
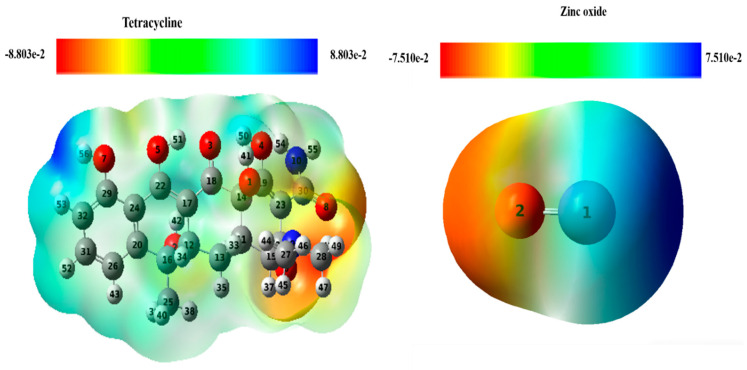
Molecular electrostatic potential maps for tetracycline and zinc oxide.

The Molecular Electrostatic Potential (MEP) map was used to analyze charge distribution and photophysical properties, helping to identify regions susceptible to nucleophilic and electrophilic attacks [24]. Figure 13 shows MEP charts for TC and zinc oxide, with red indicating electron-rich sites (susceptible to electrophilic attack) and blue representing electron-poor sites (prone to nucleophilic attack) [25,26].

For TC, electron-rich sites are located near the dimethylamine group, making them susceptible to electrophilic attacks. Conversely, electron-poor sites are found near the OH group. In zinc oxide, electron-rich sites are located near the oxygen atoms, while electron-poor sites are adjacent to the Zn atom. This means that the dimethylamine group preferentially adsorbs onto the Zn atom on the ZnO surface, while the −OH molecule adsorbs onto the oxygen atoms of this surface.

These results suggest that both molecules can interact with each other due to the presence of electrophilic and nucleophilic sites.

#### 2.7.4. Non-Covalent Interaction Analysis

**Figure 14:**
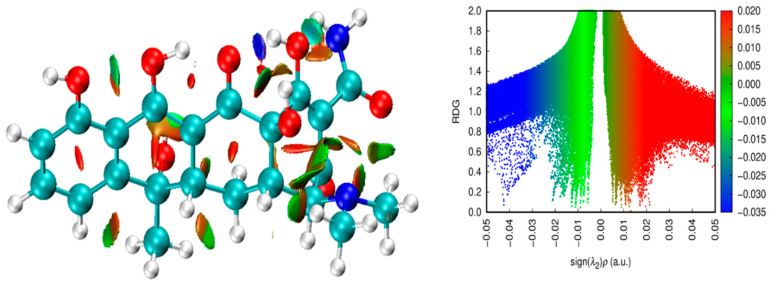
Non-covalent interaction (NCI) and reduced density gradient (RDG) diagrams for tetracycline.

We employed non-covalent interaction (NCI) diagrams generated using the Multiwfn program to examine the intermolecular interactions. These diagrams highlighted weak forces such as steric hindrance, van der Waals interactions, and hydrogen bonding, providing valuable insights into the degree of engagement between the dopant and the surface [27]. The NCI diagrams offer a detailed view of the interactions within the TC molecule, often called weak forces, and include steric hindrance, van der Waals forces, and hydrogen bonding. Figure 14 shows the NCI and reduced density gradient (RDG) diagrams for TC.

The blue regions correspond to hydrogen bonding. The strong attraction of these bonds leads to a decrease in electron density λ_2_(ρ), while repulsion results in an increase in λ_2_(ρ). RDG analysis, as developed by Johnson et al. [27], was employed to evaluate the type of NCIs present in TC. This analysis uses the sign of λ_2_ and a set of colors to indicate the nature and strength of the NCIs: sign(λ_2_)ρ < 0 indicates hydrogen bonding (blue), sign(λ_2_)ρ close to zero signifies van der Waals interactions (green), and sign(λ_2_)ρ > 0 suggests steric effects (red) [28].

Interactions were categorized within a range of −0.035 to 0.02 on a color scale. Red represents destabilizing interactions, blue denotes stabilizing regions, and green indicates weak interactions. A blend of colors depicts mixed interactions. Scatterplot colors were determined based on iso-surface values ranging from −0.05 to 0.05 atomic units, with distribution along the x-axis determined by the sign of (λ_2_)ρ.

As depicted in Figure 14, the type and strength of the NCIs can be assessed by analyzing electron density as a function of sign(λ_2_)ρ. The RDG analysis reveals that blue regions correspond to hydrogen bonding, green regions to van der Waals interactions, and red regions to steric effects. Mixed interactions appear as a blend of colors, showing the complex interaction landscape within TC.

## 3. Materials and Methods

### 3.1. Materials and Instrumentation

Zinc (II) chloride (ZnCl_2_) was acquired from Gainland Chemical Company (Flintshire, UK). Tetracycline (TC, C_22_H_24_N_2_O_8_) was purchased from Amresco (Solon, OH, USA). Ethanol (EtOH, C_2_H_6_O, 99.5%) was obtained from Merck (Darmstadt, Germany). All solutions and suspensions were prepared using deionized water (D.H_2_O, 18.2 µΩ cm^−1^). *T. vulgaris* leaves were collected from the southern Jordan area. The Department of Botany, Faculty of Science, Mutah University, Al-Karak, Jordan confirmed the taxonomy.

The pH of the solutions was measured using a pH meter (HANNA instruments, HI5521-02). An orbital shaker (LAUDA, Königshofen, Germany) was employed to shake solution contents at room temperature. A hot plate (Bibby Scientific HB502, Stone, UK) was used to heat the solutions. A Perkin-Elmer Model Lambda 25 UV–vis spectrophotometer was utilized to monitor the residual TC in the content in the solution at λ_max_ = 280 nm.

The structural and phase purity of ZnO nanoparticles was determined by X-ray powder diffraction (XRD). Using a Cu Kα radiation (λ = 1.5406 Å) source at 40 kV and 30 mA, as well as a scan rate of 2°/min, the Panalytical X’Pert Pro equipment (Malvern Panalytical GmbH, Kassel, Germany) via CuK irradiation (λ = 1.5406 Å).

Scanning Electron Microscopy (SEM) was used to study their morphology. Thermo ScientificTM Quattro ESEM (ThermoFisher Scientific, Paisley, UK) was used for SEM with an enhanced voltage of 15 kV.

### 3.2. General Protocol for T. vulgaris Extract

The freshly collected *T. vulgaris* was washed with tap water followed by D.H_2_O to remove impurities. The sample was shade-dried, ground to powder, and stored at ambient temperature. *T. vulgaris* extract was prepared in a conical flask by mixing 20 g of the plant powder with 200 mL of boiled D.H_2_O for 90 min. The mixtures were allowed to reach ambient temperature, filtered through Whatman No. 1 filter paper, and centrifuged for 30 min at 4000 rpm. The final extract was stored at 4 °C for further studies [9].

### 3.3. Fabrication of ZnO NPs

**Figure 15:**
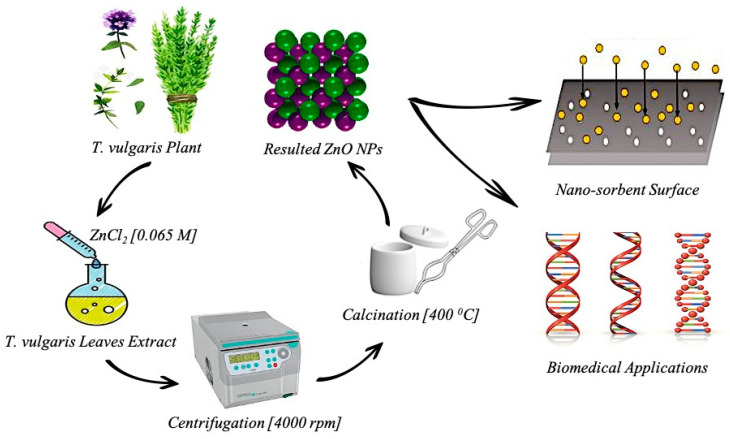
Biosynthesis steps for zinc oxide nanoparticles and their potential applications.

ZnO NPs were prepared in a round-bottom flask, by adding 5 mL of ZnCl_4_ (0.065 M) dropwise to 5 mL of *T. vulgaris* leaf extract while stirring (250 rpm) and heating to 90 °C. The pH was adjusted to 12 using NaOH (0.1 M) and HCl (0.1 M). The mixture was allowed to settle, then washed several times with hot D.H_2_O while stirring for 3 min. It was then centrifuged for 20 min at 4000 rpm and washed again with D.H_2_O and EtOH (1:1). The resulting ZnO NPs were calcined at 400 °C for 3 h, ground to powder, and stored for subsequent experiments (Figure 15).

### 3.4. Adsorption Experiments Design

The TC standard solutions were prepared by weighing 0.10 g of TC and dissolving it in 1 L of D.H_2_O. The stock solution was appropriately diluted with D.H_2_O to generate the desired 1–50 ppm concentrations. Adsorption of TC onto the prepared ZnO NPs was performed under controlled conditions. Batch adsorption experiments were conducted by mixing various amounts of ZnO NPs with TC solution in different ratios (*w*/*w*%). The operational parameters affecting TC adsorption, including adsorbent dosage (g), pH, contact time (min), temperature (°C), and initial TC concentration (ppm), were investigated.

The treatment process was performed under controlled conditions. The Erlenmeyer flasks were tightly wrapped with aluminum foil to avoid light effects throughout the TC adsorption experiments. The adsorbents and TC were added to 50 mL of aqueous media and shaken at 250 rpm in an orbital shaker until equilibrium was established. Solutions of 0.1 M HCl and 0.1 M NaOH were used to adjust and maintain the initial pH of the solutions. The NPs containing adsorbed TC were separated via a micro-filter membrane (0.22 μm). The residual TC was analyzed in the supernatant using a UV-vis spectrophotometer at λ_max_ = 280 nm.

The TC adsorption efficiency and adsorption capacity (*q_e_*) of all adsorbents were calculated using Equations (9) and (10). The mass of the ZnO NPs (*W*), the aqueous media volume (*V*), the starting concentration of TC solution (*C_i_*), and the ultimate concentration of TC (*C_f_*) were used in the calculations.

(9) $$\mathrm{Removal}\;\mathrm{Efficiency}\;(\%)=\frac{\left(C_{i}-C_{t}\right)}{C_{i}}\times 100$$

(10) $$q_{e}(\mathrm{mg}/g)=\frac{(C_{i}-C_{t})\times V}{W}$$

### 3.5. Methodology Validation

Quality assurance of the results obtained in TC residue analysis in samples can be measured in terms of external calibration and linearity limit (R^2^), detection limit (LOD, Equation (11)), limit of quantitation (LOQ, Equation (12)), precision of the modified procedure, and verification of the presented results. Additionally, the recovery rates (Recovery %, Equation (13)) of TC can be considered a validation measure for the quality of our procedure. Here, σ represents the standard deviation (SD) for the blank sample (without adsorbent), and S is the slope of the calibration curve for TC.

(11) $$\mathrm{LOD}=3.3(\frac{\sigma }{S})$$

(12) $$\mathrm{LOQ}=10(\frac{\sigma }{S})$$

(13) $$\mathrm{Recovery}\;\mathrm{rate}\;(\%)=\left(\frac{\mathrm{Amount}\;\mathrm{of}\;\mathrm{TC}\;\mathrm{recovered}}{\mathrm{Amount}\;\mathrm{of}\;\mathrm{TC}\;\mathrm{initially}\;\mathrm{present}}\right)\times 100\%$$

### 3.6. Computational Statistics

To investigate the mechanism of action between TC and zinc oxide, we employed density functional theory (DFT). DFT has become the leading method for exploring the electronic and chemical spectroscopic characteristics in quantum mechanics [29]. This study, grounded in quantum mechanical principles, presents molecular structures visualized using Gauss View 6.0.16. All computational models were theoretically evaluated using Gaussian 09 software [30].

The geometric parameters for all configurations were determined using the DFT method, employing the B3LYB functional and a 6–31 G (d, p) basis set. This basis was selected for its proven reliability in the literature for molecules of this type [31,32]. This configuration was deemed optimal for comprehensively examining the molecules, especially for evaluating their optical properties and geometric attributes.

To further explore the relationships between the compounds’ different components, topological analyses were conducted using Visual Molecular Dynamics (VMD) and Multiwfn programs [33,34]. This approach relies on minimizing non-covalent interaction density (NCI-RDG) calculations to improve the detection of interactions within the crystal structure.

## 4. Conclusions

This study highlights the green catalytic synthesis of ZnO NPs using *T. vulgaris* leaf extract. The produced ZnO nanoparticles were characterized by XRD and SEM analysis. The synthesized ZnO NPs demonstrated enhanced removal of TC from aqueous systems, with a maximum removal efficacy of 95%. Experimental investigations were conducted to optimize various parameters affecting TC adsorption onto ZnO NPs. The adsorption followed a pseudo-2nd-order kinetic model and was best described by the Temkin isotherm model. Thermodynamic data indicate that the adsorption process was spontaneous, endothermic, and increased in disorder. Overall, the green synthesis approach using plant extract-derived ZnO NPs presents a promising avenue for further research into developing ZnO nanoparticles with tailored properties for water treatment and biomedical applications. The results from DFT calculations align well with experimental findings.

## Figures and Tables

**Table 1:** Thermodynamic parameters for tetracycline adsorption using ZnO NPs.

Nanosorbent	ΔG° (KJ/mol)	△S° (1/KJ mol)	△H° (KJ/mol)
ZnO NPs	−6.42	23.79	0.55
	−6.74	-	-
	−6.92	-	-
	−7.06	-	-
	−7.16	-	-

**Table 2:** Kinetic parameters for tetracycline adsorption using ZnO NPs.

Kinetic Model	Parameter	Nanosorbent
		ZnO NPs
	q_e, exp_ (mg/g)	41.840
Pseudo-1st order	q_e, cal_ (mg/g)	0.748
	k_1_ (1/min)	0.000043
	R^2^	0.121
Pseudo-2nd order	q_e, cal_ (mg/g)	9.302
	k_2_ (g/min.mg)	1.628
	R^2^	0.9998

**Table 3:** Isotherm parameters for tetracycline adsorption using ZnO NPs.

Isotherm Model	Parameter	Value
Langmuir	q_m_ (mg/g)	294.118
	K_L_ (L/mg)	0.037
	R_L_ (dimensionless)	0.728
	R^2^	0.514
Freundlich	K_F_ (mg^1−1/n^·g^−1^·L^−1/n^)	0.083
	1/n	1.1489
	R^2^	0.840
Temkin	B_T_ (J/mol)	10.845
	K_T_ (L/mg)	2.260
	R^2^	0.918

**Table 4:** Global reactivity descriptors for tetracycline and zinc oxide.

Parameter	Tetracycline	Zinc Oxide
LUMO (eV)	−2.73	−4.09
HOMO (eV)	−5.50	−6.63
$E_{gap}$$=\mathrm{Abs}\;(E_{HOMO}$$-E_{LUMO}$) (Energy gap) (eV)	2.77	2.53
Ionization energy [I = −EHOMO]/eV	2.73	4.09
Electron Affinity [A = −ELUMO]/eV	5.50	6.63
Chemical Hardness η = (I − A)/2]/eV	−1.38	−1.26
Chemical Potential [u = −(I + A)/2]/eV	−4.11	−5.36
Softness of Molecule (s = I/2 η]/eV	−0.98	−1.61
Electronegativity [x = I + A)/2]/eV	4.11	5.36
Electrophilicity Index (ω = u2/2η]/eV	−6.11	−11.33

## Data Availability

The original contributions presented in the study are included in the article, further inquiries can be directed to the corresponding authors.
